# LncRNA‐PCAT1 targeting miR‐145‐5p promotes *TLR4*‐associated osteogenic differentiation of adipose‐derived stem cells

**DOI:** 10.1111/jcmm.13892

**Published:** 2018-10-19

**Authors:** Lingjia Yu, Hao Qu, Yifeng Yu, Wenjing Li, Yu Zhao, Guixing Qiu

**Affiliations:** ^1^ Department of Orthopaedic Surgery Peking Union Medical College Hospital Peking Union Medical College and Chinese Academy of Medical Science Dongcheng District Beijing China; ^2^ Department of Orthopaedic Surgery Beijing Jishuitan Hospital Fourth Clinical Medical College of Peking University Xicheng District Beijing China

**Keywords:** lncRNA‐PCAT1, miR‐145‐5p, osteogenic differentiation, *TLR4*, Toll‐like receptor signalling pathway

## Abstract

This study was aimed to explore the differential expression of long noncoding RNAs (lncRNA)‐PCAT1, miR‐145‐5p and *TLR4* in osteogenic differentiation via the Toll‐like receptor (TLR) signalling pathway and consequently determine the potential molecular mechanism. The mRNAs and pathways related to the osteogenic differentiation in human adipose‐derived stem cells (hADSCs) were analysed by bioinformatics. The MiRanda and TargetScan database were employed to detect the potential binding sites of miRNAs on lncRNAs and mRNAs. The differential expression of lncRNA‐PCAT1, miR‐145‐5p and *TLR4* were detected by qRT‐PCR. Rrelated protein expression was analysed by Western blot. The targeted relationships between lncRNA‐PCAT1, miR‐145‐5p and *TLR4* were verified by dual‐luciferase reporter assay. Alkaline phosphatase (ALP) activity and ARS staining assays were used to measure the impacts exerted by lncRNA PCAT1, miR‐145‐5p and *TLR4* mRNA on osteogenic differentiation. After the induction of osteoblast differentiation, the expression of lncRNA‐PCAT1 and *TLR4* increased, while the expression of miR‐145‐5p decreased. Dual‐luciferase reporter assay confirmed the targeted relationship between lncRNA‐PCAT1, miR‐145‐5p, and *TLR4*. LncRNA‐PCAT1 negatively regulated miR‐145‐5p and positively regulated *TLR4*. Knockdown of lncRNA‐PCAT1 or *TLR4* decreased the expression of osteogenic differentiation‐related proteins, reduced the ALP and ARS levels and the activity of the TLR signalling pathway. MiR‐145‐5p could reverse the effects of PCAT1 and *TLR4* in hADSCs osteogenic differentiation. LncRNA‐PCAT1 negatively regulated miR‐145‐5p, which promoted *TLR4* expression to promote osteogenic differentiation by activating the TLR signalling pathway.

## 1 INTRODUCTION

Human adipose‐derived stem cells (hADSCs) are regenerative cells locating in adipose tissue, with multilineage differentiation potentials.^1^ hADSCs can facilitate the metabolism and maintenance of tissues through adipocytes replacement and promote angiogenesis.^2^, ^3^ As an important cell type, they possess great potential to be applied to cell‐based therapies. Recent studies have indicated that hADSCs can serve as an alternative cell source to birth bone marrow mesenchymal stem cells (BMSCs), the cell source for bone regeneration.^4^ The proliferative abilities and osteogenic differentiation capabilities of BMSCs decreased with age and osteoporosis, whereas hADSCs showed consistent osteogenic differentiation capability despite of old age and osteoporotic conditions.^5^, ^6^ Therefore, the molecular mechanism related to the osteogenic differentiation of hADSCs has aroused more and more attentions.

Accumulated studies have demonstrated a significant role for long noncoding RNAs (lncRNAs) in regulating gene expression, including a significant influence on biological activities in the skeletal system, such as in osteoporosis and osteoarthritis.^7^ For example, lncRNA TUG1 promoted the differentiation of osteoblast by sponging miR‐204‐5p, which led to the up‐regulation of *Runx2*.^8^ LncRNA MEG3 restrained the osteogenic differentiation of BMSCs by targeting miR‐133a‐3p.^9^ Some studies also revealed that lncRNAs acted as epigenetic regulators in the osteo‐adipogenic lineage commitment because of the reciprocal relationship between osteogenic and adipogenic differentiation.^10^ LncRNA MIAT knockdown promoted the osteogenic differentiation of hADSCs and reversed the negative effects of inflammation.^11^ PCAT1 is a lncRNA related to the proliferation and metastasis of osteosarcomas.^12^ It acts as an oncogene with extremely high expression in osteosarcoma tissues.^13^ However, its association with the osteogenic differentiation of hADSCs remains unclear.

MiR‐145 is a microRNA (miRNA) that is broadly expressed in germline and mesoderm‐derived tissues.^14^ Several studies have indicated that it is closely related to the bone formation and differentiation. The stable expression of miR‐145 inhibits osteoblastogenesis and bone regeneration because it decreases the levels of Cbfb, a *Runx2* co‐transcription factor.^15^ Osteogenic differentiation was suppressed by miR‐145 as it negatively regulates the expression of *Sp7*, which is a transcription factor in the coordinated network essential for osteogenesis.^16^ The expression of miR‐145 was also down‐regulated in osteosarcoma compared with normal osteoblast cell lines.^17^ MiR‐145 mainly exhibited a negative effect on osteogenic differentiation.

Toll‐like receptors (TLRs) are one of the largest and best studied families of pattern recognition receptors and are related to the Toll proteins.^18^ A previous study showed that osteoblasts can express the mRNAs encoding *TLR2* and *TLR4*.^19^ *TLR4* activation can enhance both angiogenesis and osteogenesis through the coculture system of outgrowth endothelial cells and primary osteoblasts.^20^ The dysregulation of *TLR4* stands a good chance of affecting osteogenic differentiation. The associations between the TLR signalling pathway and osteogenic differentiation also remain unclear and need to be further studied.

In this study, we investigated the differential expression of mRNAs, lncRNAs and pathways in osteogenesis through bioinformatics analysis. LncRNA PCAT1 and *TLR4* were up‐regulated, and miR‐145‐5p was the common target miRNA of them. PCAT1 was found to promote the osteogenic differentiation of hADSCs by sponging miR‐145‐5p, which indirectly up‐regulated *TLR4* and activated the TLR pathway. Our discoveries indicated that lncRNA PCAT1 plays a crucial role in facilitating the osteogenic differentiation of hADSCs.

## 2 MATERIALS AND METHODS

### 2.1 Clinical samples

The study group consisted of eight osteoporosis patients who had undergone an iliac bone graft procedure during surgery at Peking Union Medical College Hospital, Peking Union Medical College and Chinese Academy of Medical Science in 2016. All patients provided informed consent and the present study was approved by the ethics committee of the Peking Union Medical College Hospital, Peking Union Medical College and Chinese Academy of Medical Science. Eight normal donors were used as controls and none of them had previously experienced bone fractures before. A standardized clinical evaluation was performed to exclude possible comorbidities.

### 2.2 Microarray analysis

Gene Expression Omnibus (https://www.ncbi.nlm.nih.gov/geo/) provided the gene expression data derived from four different donors (GSE89330). To ascertain those genes that had higher chances of being differentially expressed in one group compared with another, we used the statistical test in the R Limma package. Those genes with at least an absolute value of 1 for log_2_ (FC) and adj. *P* < 0.05 cut‐off were identified. The overall profile was normalized for each array to correct for systematic data bias and remove nonbiological impact.

### 2.3 Pathway analysis

Gene set enrichment analysis (GSEA) tested whether a set of predefined genes, usually united by shared association, showed enriched in expression levels. In our study, the uploaded gene set consisted of normalized expression data of mRNAs expression data and was sorted by the mean log_2_ signal ratios. The aggregated distribution of gene expression levels in pathways was then determined with a normalized enrichment score, which represented the statistical significance through enrichment analysis. Pathways that were significantly biased in the undifferentiated or osteogenesis group were identified. The GSEA enrichment plot offered us with visualized results.

### 2.4 DEG correlation network analysis

By evaluating the correlation between gene expression patterns, we hoped to discover potential drug targets and candidate biomarkers. The “Pearson” approach in the R package Psych was harnessed to identify linear correlations among DEGs in a specific pathway, this was adjusted by the “BH” method. Then, the networks were visualized using Cytoscape software. Nodes in the network represented DEGs while the edges indicated the presence of coexpression.

### 2.5 Cell cultivation

Human adipose‐derived stem cells were purchased from the ScienCell Company (Carlsbad, CA, USA). The cells were cultured in growth medium consisting of Dulbecco's Modified Eagle's Medium (DMEM; Gibco, New York, NY, USA) supplemented with 10% foetal bovine serum (FBS) and 1% antibiotics (Gibco). All cell‐based in vitro experiments were performed in triplicate.

### 2.6 Osteoblast differentiation induction

To induce osteoblast differentiation, the media was replaced with osteoblasts‐specific induction medium upon reaching 80%‐90% confluence (cells covered area percentage). Low glucose of DMEM with 10% FBS, 10 mM β‐glycerophosphate, 0.1 μM dexamethasone, and 0.2 mM antiscorbic acid (Sigma‐Aldrich, Louis, MO, USA) was useful for inducing osteoblast differentiation for 0, 7, 14, and 21 days.

### 2.7 Cell proliferation dynamics analysis

Passage 4 cells were seeded into 96‐well plates at 6.25 × 10^4^, 1.25 × 10^5^, 2.5 × 10^5^, 5 × 10^5^, 1 × 10^6^ and 2 × 10^6^ cells/well cell concentration, respectively. Taking the cell density as the *X*‐axis, the absorbance as the *Y*‐axis, draw the standard curve. Cell proliferation was detected by using the Cell Counting Kit‐8 kit (Beyotime, Shanghai, China). Cells were seeded into 96‐well plates at 2 × 10^3^ cells/well cell concentration. After cell culture for 1‐21 days, 10 μL CCK‐8 solution was added to each well and incubated at 37°C for 2 hours. The absorbance was measured at a wavelength of 570 nm for each well. The population doubling was calculated according to the standard and growth curves. Population doubling *T*_*d*_ = *T* × [lg2/lg(*N*_*t*_/*N*_0_)]. *T*, proliferation time; *N*_0_, cell inoculum density; *N*_*t*_, late cell density.

### 2.8 hADSC phenotypic characterization

hADSC‐specific surface antigens were stained with PE‐conjugated antibodies of anti‐human CD29 (BD, Franklin Lakes, NJ, USA), anti‐human CD34 (BD), anti‐human CD44 (BD), and anti‐human CD45 (BD) or their corresponding isotype control. Then, the stained cells were analysed by fluorescence‐activated cell sorting (FACS). In brief, hADSCs were separately by trypsin‐EDTA and gently blown into single cells, which were fixed and permeated with a Cytofix/Cytoperm Fixation/Permeabilization Solution Kit (BD) following the manufacturer s instructions. After stained with PE‐conjugated antibodies for 30 minutes at 4°C, these stained cells were analysed on a fluorescence‐activated cell sorter (Beckman, Miami, FL, USA). The experiments were repeated three times, the results are presented as fold change ± SD, and *P* < 0.05 was considered significantly different.

### 2.9 QRT‐PCR

After osteoblast differentiation was induced in cells for 14 days, the cells were dissociated with 0.25% trypsin with EDTA and washed twice with cold PBS. The cell suspension was centrifuged at 111.9 × *g* for 5 minutes to get the precipitation. RNA extraction was performed using the Takara Minibest universal RNA extraction kit (Takara, Katsushika, Tokyo) according to the manufacturer's protocol. In total 500 ng of total RNA was reverse‐transcribed to cDNA using the Takara PrimeScript RT master mix kit (Takara) according to the manufacturer's instructions. MiRNAs were reverse‐transcribed with an All‐in‐OneTM miRNA first‐strand cDNA synthesis kit (GeneCopoeia, Rockville, MD, USA). Equivalent amounts of cDNA were used for real‐time PCR in a 20 μL reaction mixture using SYBR Premix Ex TaqII kit (catalog number: RR820A; Takara). QRT‐PCR reactions were performed with 40 cycles of amplification on an ABI Prism 7300 Real‐Time PCR system (Applied Biosystems, Waltham, MA, USA). The primers (Invitrogen, Waltham, MA, USA) used are listed in Table S1. The results were evaluated by the 2^−ΔΔCt^ method.

### 2.10 Western blot

After osteoblast differentiation was induced in cells for 14 days, the cells were washed twice with PBS and harvested in 200 μL lysis buffer (100 μM HEPES, 10 mM KCl, 2 mM MgCl_2_, and 1 mM DTT) containing complete protease inhibitor (Roche Diagnostics Deutschland GmbH, Mannheim, Germany). Five microliters 10% Nonidet‐P40 was added, followed by centrifugation at 18911.1 × *g* for 30 seconds at 4°C. The Bradford protein assay (Bio‐Rad, Hercules, CA, USA) was used to quantify the protein concentration. Samples were separated by SDS polyacrylamide gel electrophoresis and then transferred to a PVDF membrane using the iBlot Dry Blotting Transfer System (Life Technologies Corporation, Gaithersburg, MD, USA). Membranes were blocked with 2.5% nonfat milk powder in TTBS buffer (0.1 M Tris, 150 mM NaCl, and 0.1% Tween‐20) and incubated with primary antibodies against TLR4 (1/500, ab13556; Abcam, Invitrogen, OR, USA), ERK1/2 (1/1000, ab17942; Abcam), p‐ERK1/2 (1/1000, ab214362; Abcam), JNK (1/1000, ab179461; Abcam), p‐ JNK (1/1000, ab124956; Abcam), RUNX2 (1/1000, ab23981; Abcam), OPN (1/1000, ab8448; Abcam), OCN (1/500, ab93876; Abcam), primary antibodies overnight at 4°C. Membranes were washed three times for 15 minutes with TTBS followed by incubation for 2 hours at room temperature with secondary antibodies. After three more washes for 15 minutes with TBST, specific staining was detected using the chemiluminescence (ECL) system (VWR International GmbH, Darmstadt, Germany). All bands were densitometrically analysed with ImageJ.

### 2.11 Cell transfection

The miR‐145‐5p mimics, si‐PACT1 and si‐TLR4 were synthesized by Genomeditech (Shanghai, China). Oligonucleotide transfection was conducted using Lipofectamine 2000 transfection reagent (Invitrogen) following manufacturer's recommendations. Forty‐eight hours after transfection, the cells were collected for further investigations.

### 2.12 Alkaline phosphatase staining

The expression of alkaline phosphatase (ALP) in the cell layers was assessed using a kit according to the manufacturer's instructions (Yeasen, Shanghai, China). Fourteen days after osteogenic induction, the hADSCs were rinsed three times with PBS three times and fixed with 70% alcohol for 30 minutes. The fixed cells were soaked in BCIP/NBT solution, washed with ddH2O, and then observed with a scanner (ImageScanner III, GE Healthcare Bio‐Sciences Corp., Piscataway, NJ, USA). Each assay condition was repeated in triplicate. All the results were repeated in three independent experiments.

### 2.13 Alizarin red S staining

Alizarin red S staining was used to detect matrix mineralization. Fourteen days after osteogenic differentiation, the cultured cells were treated with 75% ethanol for 20 minutes, and then stained with 1% Alizarin Red S pH 4.2 (ShangHai Haoran Biological Technology, Shanghai, China) for 1 hour at 37°C for the semi‐quantitative evaluation of the degree of mineralization.

### 2.14 Dual‐luciferase reporter assay

The PCAT1 and TRL4 sequences containing the predicted potential miR‐145‐5p binding sites were amplified. The PCAT1‐WT and TRL4‐WT plasmids were constructed using a PCR method. PCAT1‐MUT and TLR4‐MUT were generated by site‐directed mutagenesis, replacing the first six ribonucleotides of the miR‐145‐5p complementary sequence. HEK293T cells (BeNa Culture Collection, China) were grown in 24‐well plates and cotransfected with miR‐145‐5p mimics and PCAT1‐WT, miR‐145‐5p mimics and PCAT1‐MUT, control mimics and PCAT1‐WT, control mimics and PCAT1‐MUT, miR‐145‐5p mimics and TLR4‐WT, miR‐145‐5p mimics and TLR4‐MUT, control mimics and TLR4‐WT, control mimics and TLR4‐MUT. The media was replaced after 6 hours; 24 hours after cotransfection, the Dual‐Luciferase Reporter Gene Test Kit (Promega, Fitchburg, WI, USA) was used to detect the luciferase activities in different groups. The firefly and the renal luciferase reagent were added to detect the luciferase activity of each group.

### 2.15 Statistical analysis

Numerical data were expressed as the mean ± SD. Student's *t* test rendered statistical comparisons. The criterion for significance was set to as *P* < 0.05.

## 3 RESULTS

### 3.1 The profiling of dysregulated mRNAs, lncRNAs, and activated toll like receptor signalling pathway during osteogenic differentiation

**Figure 1:**
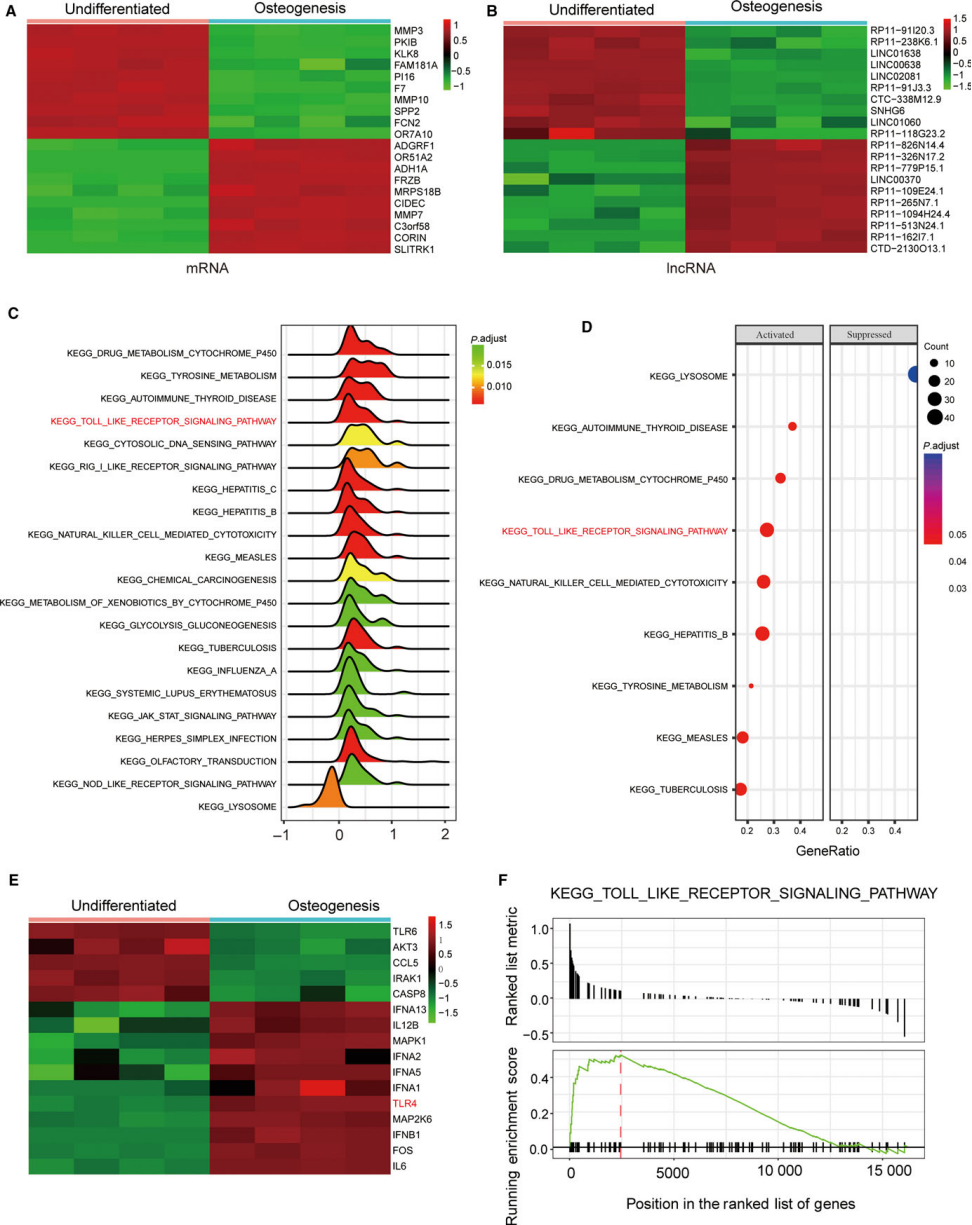
Dysregulated mRNAs, lncRNAs and activated toll like receptor signalling pathway in osteogenic differentiation. (A) Dysregulated mRNAs in osteogenic differentiation. The top ten up‐regulated and down‐regulated mRNAs were displayed in heatmap. (B) Dysregulated lncRNAs in osteogenic differentiation. The top 10 up‐regulated and down‐ regulated mRNAs were displayed in heatmap. (C) The ridgeplot results show the distributions of some significantly biased KEGG pathways; the Toll‐like receptor signalling pathway was up‐regulated in osteogenic differentiation. (D) The dotplot result show that Toll‐like receptor signalling pathway was activated in osteogenic differentiation. (E) The genes with biased expression level in Toll‐like receptor signalling pathway and *TLR4* was displayed as highly expressed in this pathway. (F) The gseaplot result indicated that most genes involved in Toll‐like receptor signalling pathway are overexpressed in osteogenic differentiation

Figure 1A and B showed 20 selected mRNAs and lncRNAs, respectively, that demonstrated the most significant differences in their expression profiles during osteogenic differentiation, with 10 ranked at the top and 10 at the bottom sorted by their fold‐change value. As shown in Figure 1C and D, the pathways which were significantly biased were demonstrated by ridgeplot and dotplot based on the GSEA analysis results. The Gseaplot result showcased that the TLR signalling pathway was activated (Figure 1F). Furthermore, *TLR4* was highly expressed in TLR signalling pathway, which was shown in Figure 1E. All results demonstrated that TLR signalling pathway was activated in osteogenic differentiation. Therefore, these results provided us with insights that led us to focus on TLR signalling pathway in the next study.

### 3.2 The putative targeted relationship between lncRNA PCAT1, miR‐145‐5p, and *TLR4*

**Figure 2:**
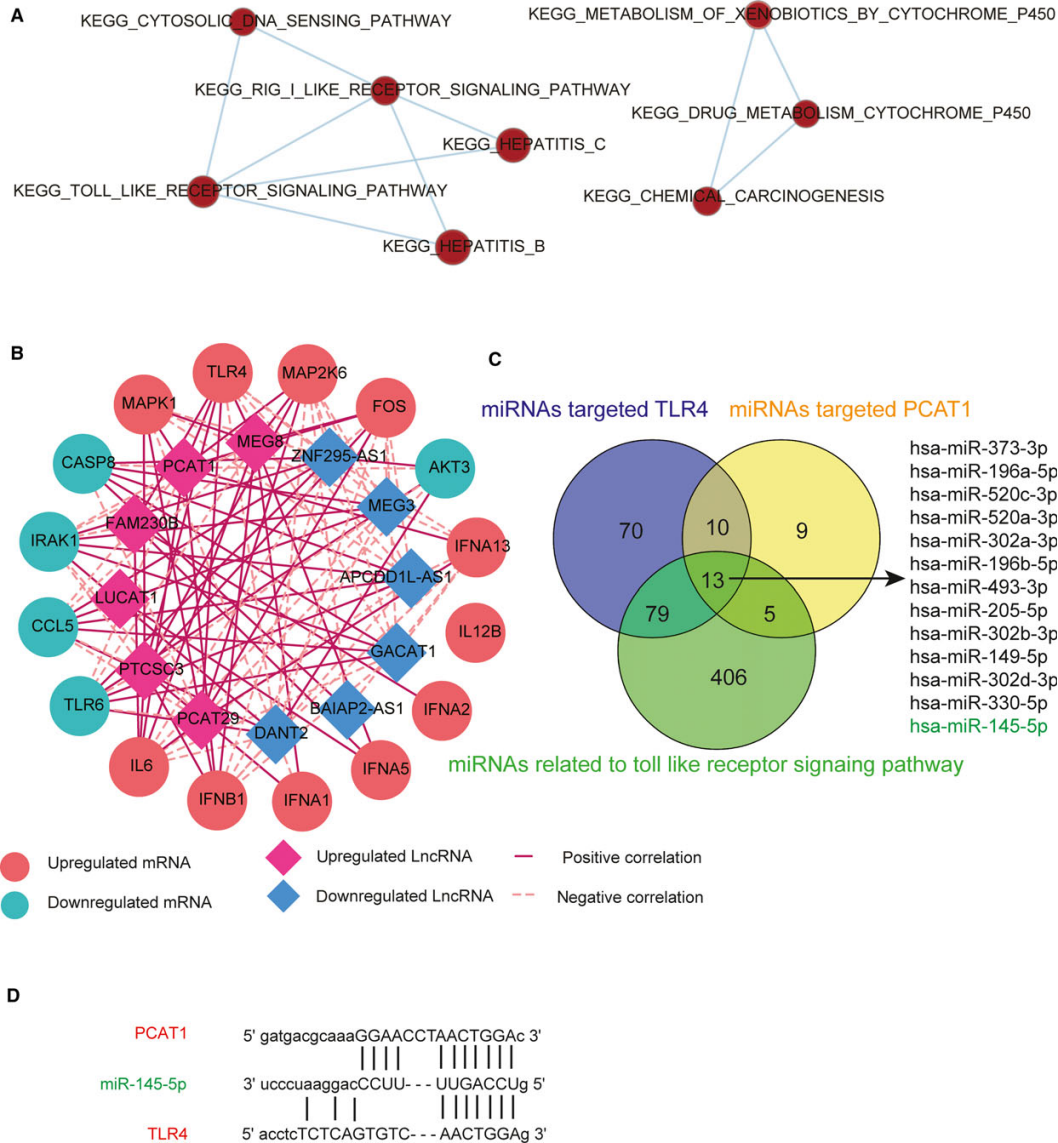
Gene coexpression network and estimated binding sites. (A) Signalling pathway enrichment map. Nodes in the map denoted specific gene sets with related biological functions and weighted links connecting different nodes represent overlap based on the of number of genes shared by two gene sets. (B) Gene coexpression network. The solid line represented the positive correlation between lncRNAs and mRNAs while the dashed line represented the negative correlation. (C) The Veen diagram shows the common miRNAs targeting *TLR4* and PCAT1 to the Toll‐like receptor signalling pathway simultaneously. (D) The PCAT1 and miR‐145 as well as miR‐145‐4p and *TLR4* binding sites and the putative binding relationship were illustrated

An enrichment map was implemented to indicate those pathways containing cross‐referencing genes. The TLR signalling pathway was marked with dark red, which denoted it was highly enriched. The connections showed overlapping parts, indicating that the TLR signalling pathway contained genes that cross‐referenced with four other pathways (Figure 2A). The gene coexpression network in Figure 2B displays the correlation relationship between lncRNA‐PCAT1 and *TLR4*. Constructed using the Pearson correlation coefficient, mRNAs contained in TLR signalling pathway and lncRNAs that were linearly related to mRNAs were screened as the nodes in the network depending on their expression level vairations. The results were visualized via Cytoscape 3.6.5. Considering all the miRNAs that targeted *TLR4*, PCAT1, and the TLR signalling pathway together, 13 miRNAs were identified by the Veen diagram. We choose miR‐145‐5p, which was included among these thirteen miRNAs, for further study (Figure 2C). The PCAT1, miR‐145 and *TLR4* binding sites as well as the putative binding relationship were illustrated in Figure 2D.

### 3.3 The morphology, propagation, and characterization of passage 4hADSCs

**Figure 3:**
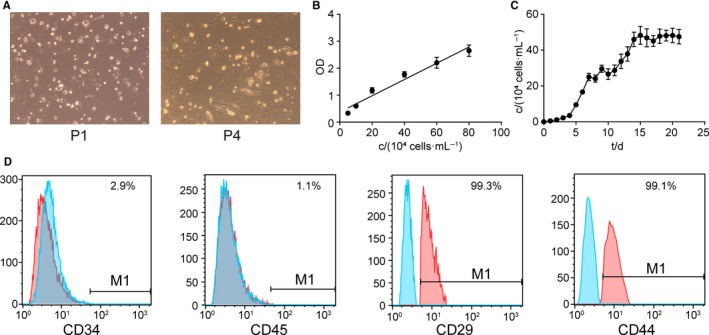
The morphology, propagation, and characterization of hADSCs was tested. (A) Morphology change of hADSCs from subcutaneous fat tissue cultured in vitro. Microscopic multiple with 100×. (B) Standard curve was gained according to cell density and absorbance. (C) Growth curves of hADSCs. (D) The expression of surface antigens (CD29, CD34, CD44, CD45) in passage 4 hADSCs was detected by flow cytometry

The seeded hADSCs began to grew by static adherence in 24 hours. The primary cells were relatively single, short shuttle‐like, or round. While after three times of passage, cell grew into spindle‐shaped, fibroblast‐like appearance cells with spherical or orbicular‐ovate nucleus under high power lens (×100), with rapid proliferation and swirl distribution (Figure 3A). A linear relationship between absorbance and density of live cells in growth period was displayed (Figure 3B). Furthermore, CCK‐8 assay was performed to detect the proliferation of hADSCs, and the growth curve was draw for population doublings calculation. As shown in Figure 3C, 4‐7 days were logarithmic phase, the population doubling was 25.38 hours calculated according to the standard curve and growth curve. In addition, FACS was used to characterize the hADSCs. Our results showed that the cell surface markers CD29 and CD44 were highly expressed and that the surface markers CD34 and CD45 were minimally expressed in hADSCs (Figure 3D).

### 3.4 The expression of lncRNA‐PCAT1, miR‐145‐5p, and *TLR4* were changed in hADSCs after Osteoblast differentiation induction

**Figure 4:**
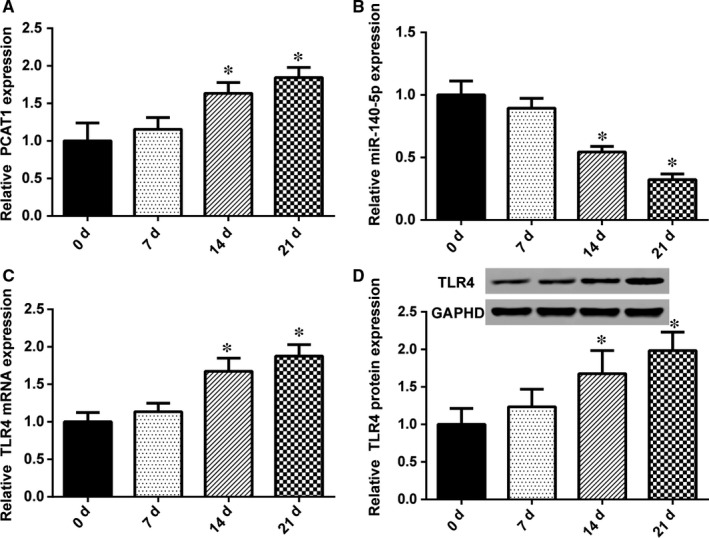
Expression of lncRNA PCAT1, miR145‐5p and *TLR4* in osteogenic differentiation. (A) LncRNA PCAT1 expression showed a significant increase until 14 and 21 days after the induction osteoblast differentiation (*P < 0.05 compared with 0 day). (B) MiR‐145‐5p was significantly lowly expressed after osteoblast differentiation induction (*P < 0.05 compared with 0 day). (C) The expression of *TLR4* was increased after osteoblast differentiation induction. On the 14th day and 21th day, it increased obviously (*P < 0.05 compared with 0 day). (D) The expression of TLR4 protein was detected by Western‐blot, and also increased after osteoblast differentiation induction, especially after 14 and 21 days (*P < 0.05 compared with 0 day)

The changes in lncRNA PCAT1, miR‐145‐5p, and *TLR4* mRNA expression in hADSCs were detected by qRT‐PCR, after the induction of osteoblast differentiation induction for 0, 7, 14, and 21 days. As shown in Figure 4A, following the induction of osteoblast differentiation, lncRNA PCAT1 expression was up‐regulated compared with 0 day, exhibiting a significant increase until 14 and 21 days after the induction of osteogenic differentiation. However, miR‐145‐5p expression showed the opposite tendency (Figure 4B); after induced, its expression decreased. Additionally, after induction for 14 and 21 days, the decrease was notable. The results for *TLR4* mRNA were similar to lncRNA‐PCAT1. Until 14 and 21 days after induced osteogenic, the expression of *TLR4* was remarkable (Figure 4C). TLR4 protein expression was also detected by Western blot (Figure 4D). TLR4 protein expression was positively correlated with mRNA expression.

### 3.5 The correlation between lncRNA‐PCAT1, miR‐154‐5p, and *TLR4* in osteoporosis

**Figure 5:**
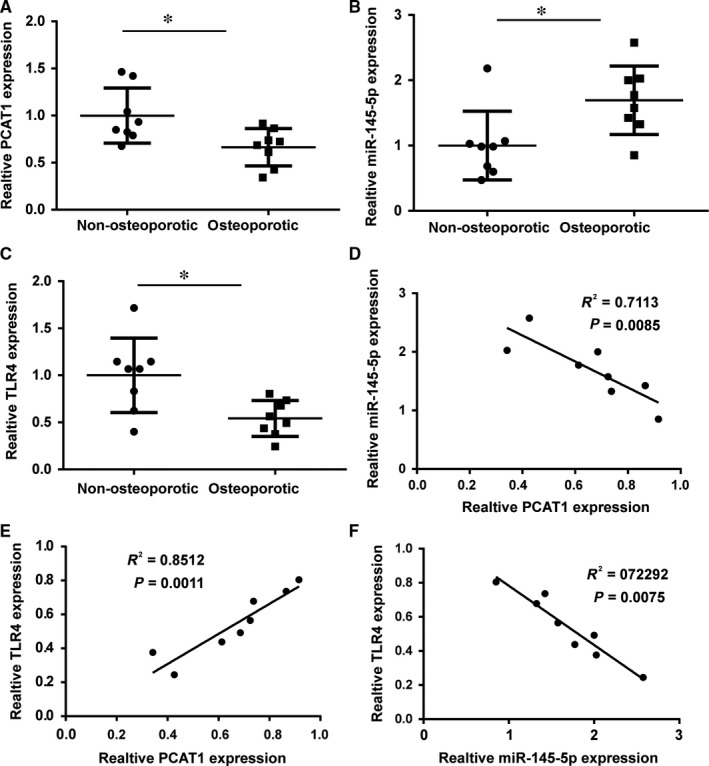
The correlation between lncRNA‐PCAT1, miR‐154‐5p, and *TLR4* in osteoporosis patient. (A) PCAT1 expression in eight nonosteoporotic samples and eight osteoporotic samples, *P < 0.05. (B) MiR‐145‐5p expression in eight nonosteoporotic samples and eight osteoporotic samples, *P < 0.05. (C) The expression of *TLR4* in eight nonosteoporotic samples and eight osteoporotic samples, *P < 0.05. (D) There is a significantly negative correlation between miR‐145‐5p and PCAT1 expression. (E) The expression of *TLR4* and PCAT1 revealed a significantly positive correlation. (F) There is a significantly negative correlation between *TLR4* and miR‐145‐5p expression

LncRNA‐PCAT1, miR‐154‐5p, and *TLR4* expression were detected in eight nonosteoporotic samples and eight osteoporotic samples. PCAT1 expression was decreased in the eight osteoporotic samples compared with the nonosteoporotic samples (Figure 5A). *TLR4* expression was also decreased in the eight osteoporotic samples compared with nonosteoporotic samples (Figure 5C). However, the expression of miR‐145‐5p revealed the opposite results. The expression of miR‐145‐5p was higher in nonosteoporotic samples (Figure 5B). Statistical analysis showed the correlation between lncRNA‐PCAT1, miR‐145‐5p, and *TLR4*. There was a significantly negative correlation between miR‐145‐5p and PCAT1 expression (Figure 5D). *TLR4* and miR‐145‐5p expression also revealed a significantly negative correlation (Figure 5F). Moreover, there is a significantly positive correlation between *TLR4* and miR‐145‐5p expression (Figure 5E).

### 3.6 The targeted relationship between lncRNA‐PCAT1, miR‐145‐5p, and *TLR4*

**Figure 6:**
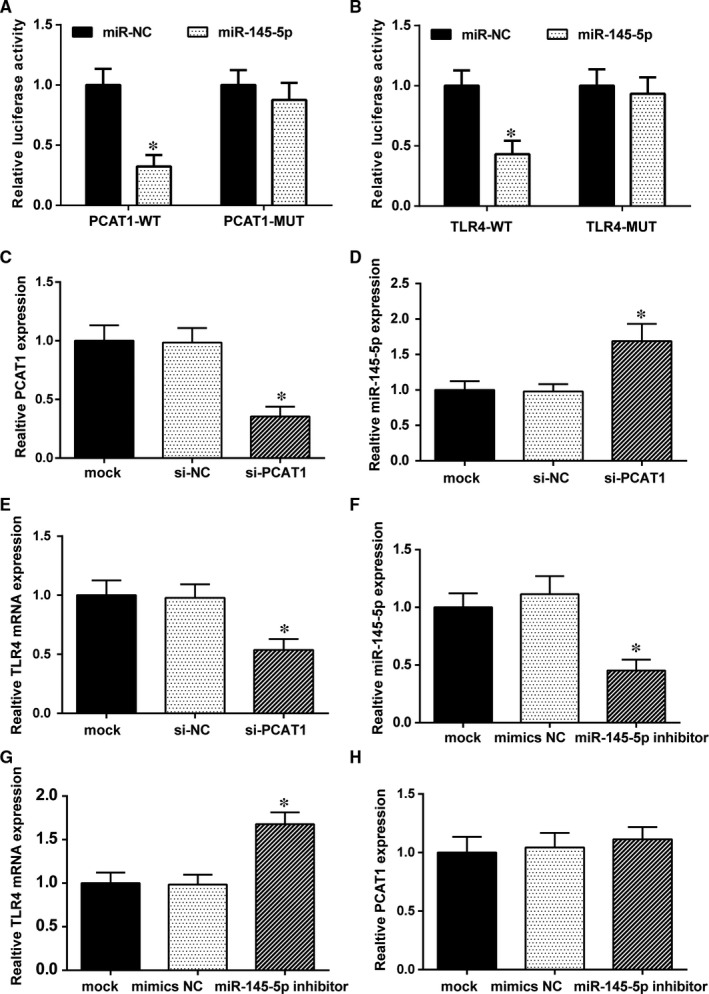
The relationship between lncRNA PCAT1, miR‐145‐5p and *TLR4*. (A) Dual luciferase reporter gene assay results demonstrated that the luciferase activity in the miR‐145‐5p mimics+PCAT1‐WT group was significantly weaker than in the miR‐NC+PCAT1‐WT group. However, there were no significant changes between miR‐145‐5p mimics+PCAT1‐MUT group and miR‐NC+ PCAT1‐MUT group (*P < 0.05 compared with the miR‐NC group). (B) The luciferase activity in the miR‐145‐5p mimics+TLR4‐WT group was much lower than in the miR‐NC group. There were no significant changes between the miR‐145‐5p mimics group and NC group after *TLR4* mutation (*P < 0.05 compared with miR‐NC group). (C) LncRNA PCAT1 expression was decreased after PCAT1 knockdown (*P < 0.05 compared with the mock group). (D) The expression of miR‐145‐5p was increased after PCAT1 knockdown (*P < 0.05 compared with mock group). (E) The expression of *TLR4* was decreased after PCAT1 knockdown (*P < 0.05 compared with the mock group). (F) MiR‐145‐5p expression was decreased after miR‐145‐5p knockdown (*P < 0.05 compared with the mock group). (G) *TLR4* expression was increased after miR‐145‐5p knockdown (*P < 0.05 compared with mock group). (H) LncRNA PCAT1 expression showed no change after miR‐145‐5p knockdown (*P* > 0.05 compared with the mock group)

The luciferase reporter gene assay was conducted in HEK293T cells for verifying their binding relationship. The result revealed that the luciferase activity of cells cotransfected with miR‐145‐5p and PCAT1‐WT was significantly inhibited, whereas miR‐145‐5p had no such impact on the PCAT1‐MUT fluorescence (Figure 6A). Similar results are shown for the relationship between miR‐145‐5p and *TLR4* mRNA (Figure 6B). The luciferase activity of cells cotransfected with miR‐145‐5p and TLR4‐WT was significantly reduced, while the luciferase activity of cells cotransfected with miR‐145‐5p and TLR4‐MUT was practically similar. These results indicated that lncRNA‐PCAT1 and miR‐145‐5p as well as miR‐145‐5p and *TLR4* mRNA directly regulated one another.

To further demonstrated the relationship between lncRNA‐PCAT1, miR‐145‐5p and *TLR4* mRNA, we transfected si‐PCAT1 plasmids and miR‐145‐5p inhibitors into hADSCs and observed the effects on PCAT1, miR‐145‐5p, and *TLR4* expression. The results revealed that si‐PCAT1 decreased PCAT1 (Figure 6C) and *TLR4* mRNA (Figure 6E) expression compared with the mock and si‐NC groups,whereas miR‐145‐5p was up‐regulated (Figure 6D). MiR‐145‐5p was decreased after the transfection of the miR‐145‐5p inhibitors (Figure 6F), while PCAT1 and *TLR4* expression showed completely different results. *TLR4* expression was increased (Figure 6G) and PCAT1 showed barely any change in expression (Figure 6H) after transfected miR145‐5p inhibitor, compared with mock and mimics NC groups. Taken together, these results revealed that lncRNA‐PCAT1 could negatively regulate miR‐145‐5p and positively regulate *TLR4*. What's more, miR145‐5p also negatively regulated *TLR4*, but did not regulate lncRNA‐PCAT1.

### 3.7 LncRNA‐PCAT1, miR‐145‐5p, and mRNA *TLR4* affected osteogenic differentiation

**Figure 7:**
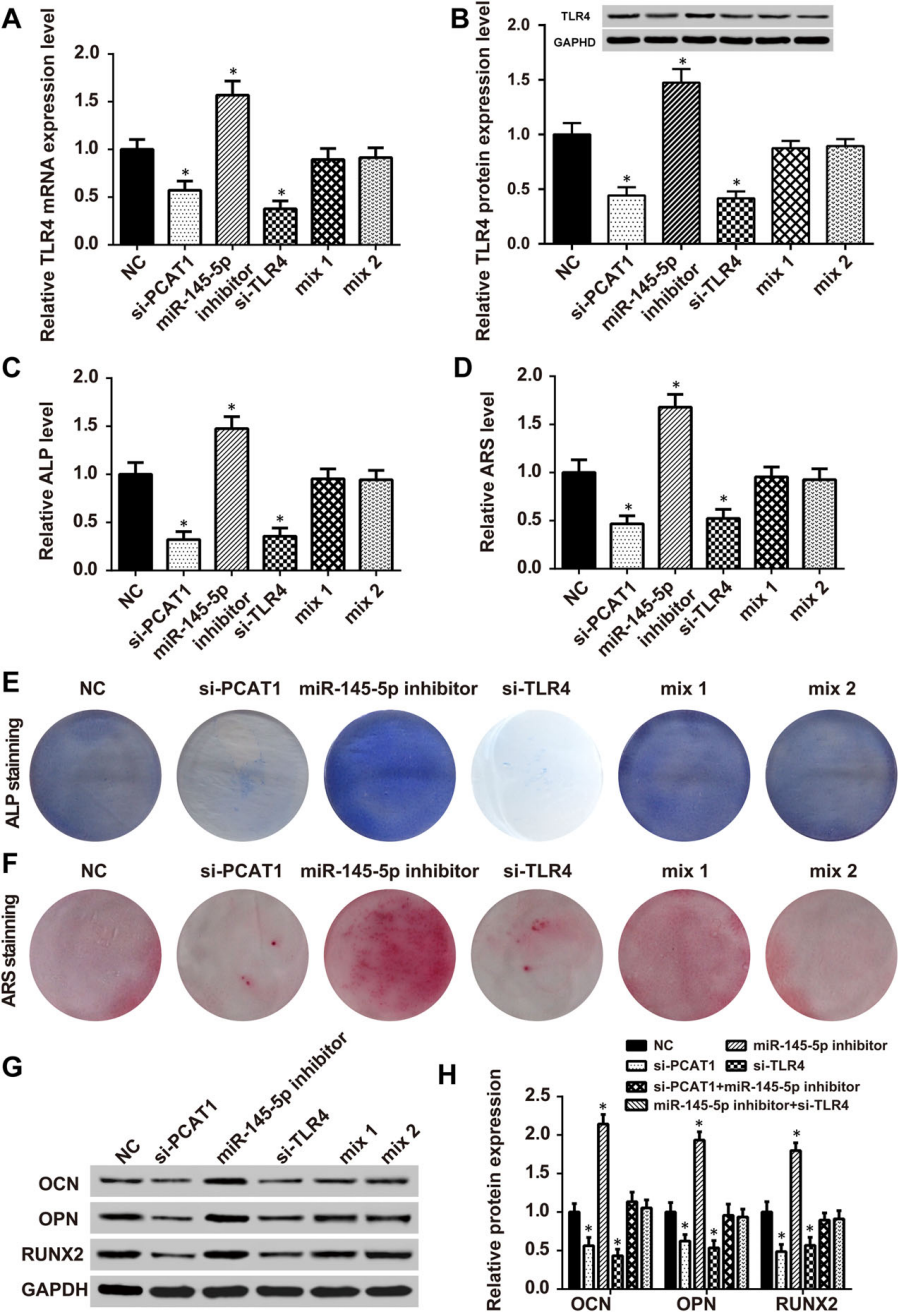
ALP and ARS staining assay results because of the impact of lncRNA PCAT1, miR‐145‐5p and *TLR4* on osteogenic differentiation. (A and B) *TLR4* mRNA and protein expression were increased after miR‐145‐5p knockdown but reduced in the si‐PCAT1 group and si‐TLR4 group. Mix1 (si‐PCAT1+ miR‐145‐5p inhibitor) and mix2 (miR‐145‐5p inhibitor+si‐TLR4) showed no significant change (*P < 0.05 compared with NC group). (C–E) ALP staining results demonstrated that knockdown of miR‐145‐5p significantly strengthened osteogenic differentiation while the transfection of si‐PCAT1 or si‐TLR4 reduced osteogenic differentiation. The simultaneous transfection of si‐PCAT1+ miR‐145‐5p inhibitor and miR‐145‐5p inhibitor+si‐TLR4 showed hardly any significant difference from the NC group alone (*P < 0.05 compared with the NC group). (D–F) ARS staining results showed similar results. MiR‐145‐5p knockdown significantly strengthened osteogenic differentiation while transfection of si‐PCAT1 or si‐TLR4 reduced osteogenic differentiation. The simultaneous transfection of si‐PCAT1+ miR‐145‐5p inhibitor and miR‐145‐5p inhibitor+si‐TLR4 showed no significant differences compared with the NC group (*P < 0.05 compared with the NC group). (G and H) OCN, OPN, and RUNX2 expression was increased after miR‐145‐5p knockdown but was reduced in the si‐PCAT1 group and si‐TLR4 groups. Mix1 (si‐PCAT1+ miR‐145‐5p inhibitor) and mix2 (miR‐145‐5p inhibitor+si‐TLR4) showed no significant changes (*P < 0.05 compared with NC group)

The transfection of si‐PCAT1 and si‐TLR4 into hADSCs led to a simultaneous in TLR4 mRNA expression level and TLR4 protein levels (Figures 7A and B). PCAT1 and *TLR4* knockdown remarkably inhibited ALP levels, while miR‐145‐5p inhibitors increased ALP levels. Mix1 and mix2 recovered ALP levels to normal. The relative ARS levels were similar to ALP levels (Figures 7C–F), Western blot detected the expression of OCN, OPN and RUNX2, which are related to osteogenic differentiation. As shown in Figures 7G and H, compared with the noninduced group, OCN, OPN, and RUNX2 expression levels decreased significantly in the si‐PCAT1 and si‐TLR4 groups, but were up‐regulated in the miR‐145‐5p inhibitor group.

### 3.8 LncRNA PCAT1, miR‐145‐5p, and mRNA *TLR4* affected Toll like receptor signalling pathway

**Figure 8:**
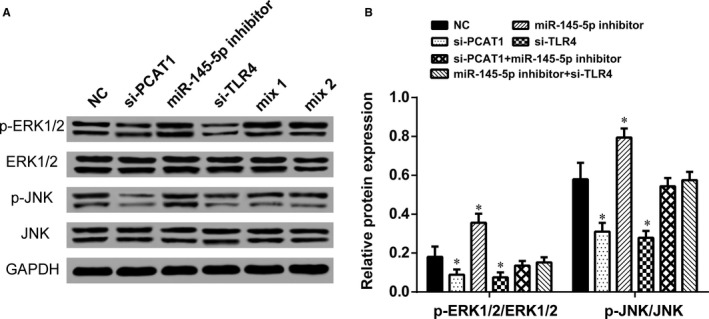
Western blot analysis showing the effect of PCAT1, miR‐145‐5p and *TLR4* on Toll‐ like receptor signalling pathway. (A) Western blot analysis of phosphorylated ERK1/2 (p‐ERK1/2), ERK1/2, phosphorylated JNK (p‐JNK), and JNK protein levels in different groups after osteogenic differentiation for 14 days (noninduced, si‐PCAT1, miR‐145‐5p inhibitor, si‐TLR4, mix1, and mix2). (B) The expression levels of the Toll‐like receptor signalling pathway downstream proteins, p‐ERK1/2/ERK1/2 and p‐JNK/JNK value were down‐regulated by PCAT1 or *TLR4* knockdown in hADSCs and up‐regulated by miR‐145‐5p knockdown. *P < 0.05, compared with NC group

**Figure 9:**
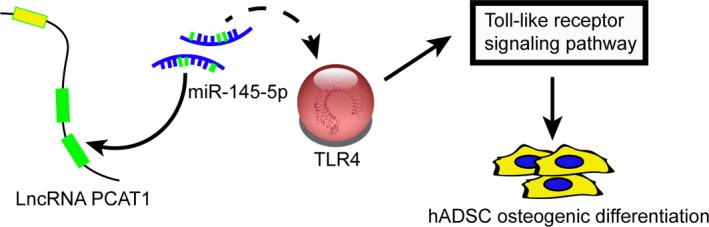
A proposed schematic diagram of LncRNA PCAT1/miR‐145‐5p/TLR4 mRNA axis during the osteogenic differentiation process. (A) We speculated that LncRNA‐PCAT1 sponged miR‐145‐5p to promote TLRA associated osteogenic differentiation of hADSCs via the activation of Toll‐like receptor signalling pathway

The expression level of p‐ERK1/2 and p‐JNK, downstream proteins in TLR signalling pathway, were observed by Western blot analysis to determine whether PCAT1, miR‐145‐5p, and *TLR4* influence this pathway. The expression of p‐ERK1/2/ERK1/2 and p‐JNK/JNK were inactivated when PCAT1 or *TLR4* was knockdown and were activated when miR‐145‐5p was knockdown (Figure 8A and B). In contrast, p‐ERK1/2/ERK1/2 and p‐JNK/JNK expression was barely changed in mix1 group and mix2 group (*P* > 0.05). Therefore, our data suggested that LncRNA‐PCAT1 sponged miR‐145‐5p to promote TLRA associated osteogenic differentiation of hADSCs via the activation of TLR signalling pathway (Figure 9A).

## 4 DISCUSSION

In the present study, we investigated the osteogenic differentiation of hADSCs, showing that lncRNA PCAT1 and *TLR4* were up‐regulated in ASC osteogenic differentiation and that the TLR signalling pathway that was activated. MiR‐145‐5p is the common PCAT1 and *TLR4* target miRNA. LncRNA PCAT1 acted as a competing endogenous RNA (ceRNA) of miR‐145‐5p in ASC to promote the osteogenic differentiation by up‐regulating *TLR4* and activating the TLR pathway.

The differentially expressed lncRNAs, mRNAs and pathways in hADSCs osteogenic differentiation were screened by microarray analysis and using the GSEA software. The expressions of lncRNA PCAT1, *TLR4* mRNA and the TLR pathway were remarkably increased in hADSCs osteogenic differentiation. PCAT1 is a lncRNA that has been extensively studied in terms of its correlation with tumour progression and prognosis.^21^ It was found to show significant biased expression in various kinds of human cancers, such as prostate cancer,^22^ glioblastoma,^23^ and gastric cancer.^24^ However, its function in the human skeletal system has not been previously emphasized. Previous studies only showed its impact on osteosarcoma, in which PCAT1 was up‐regulated and promoted osteosarcoma tumourigenicity.^12^, ^13^ Here, we found that PCAT1 positively regulated the osteogenic differentiation of hADSCs, suggesting that PCAT1 might be an important target lncRNA for ASC‐based therapies related to osteogenic differentiation.

*TLR4* was also identified to be associated with the osteogenic differentiation of hADSCs in this study. Similar to PACT1, *TLR4* promoted the osteogenic differentiation of hADSCs. Raicevic et al demonstrated a similar effect for *TLR4* in adipose tissue, as osteogenesis was increased by triggering TLR3 and TLR4 in BMSCs and adipose tissues.^25^ Although studies on the role of *TLR4* in ASC osteogenic differentiation are limited, some evidence implies that TLR4 is involved in several biological processes in the skeletal system. *TLR4* was reported to participate in the transformation of adventitial fibroblasts to myofibroblasts regulated by osteocalcin.^26^ In germ‐positive bacterial bone infections, *TLR2* and *TLR4* were related to the increased production of human beta‐defensin‐3 in osteoblasts.^27^ *TLR4* expression was up‐regulated by the overexpression of HSP60, one of the heat shock proteins that played a critical role in promoting bone loss, and silencing of *TLR4* silencing almost eliminated the HSP60 mediated promotion of BMSC apoptosis.^28^ Our findings revealed that *TLR4* greatly facilitated the osteogenic differentiation of hADSCs, providing a novel insight into *TLR4*'s biological function. Meanwhile, the TLR pathway was also activated, which was consistent with the up‐regulation of *TLR4*.

A connection between lncRNA PCAT1 and *TLR4* was established by miR‐145‐5p, which was proven to be able to regulate the osteoblastic differentiation. MiR‐145‐5p expression was decreased during osteogenic differentiation, and transfection of miR‐145 mimics in pluripotent mesenchymal precursor cells reduced the expression of osteogenic differentiation markers.^16^ The undifferentiated interaction networks of mesenchymal stem cells exhibited an increased miR‐145‐3p identification.^29^ In human osteoblast‐like MG‐63 cells, suppression of miR‐145 could stimulate the expression of osteoprotegerin, a soluble glycoprotein that reduced bone resorption and enhances bone formation.^30^ MiR‐145 also negatively affected the osteoblastic differentiation by targeting *Cbfb*, the transcription factors that are essential for bone formation.^15^ These previous studies revealed the suppressive role of miR‐145 in osteogenic differentiation, which was in line with our study as we found miR‐145‐5p suppressed the osteogenic differentiation of hADSCs.

Furthermore, miR‐145‐5p was the common target miRNA for lncRNA PCAT1 and *TLR4*, suggesting that there is a PCAT1/miR‐145‐5p/*TLR4* axis in the regulation of ASC osteogenic differentiation. LncRNA PCAT1 served as a ceRNA to sponge miR‐145‐5p and up‐regulate *TLR4*. Similar regulatory mechanisms have been identified for other lncRNAs in osteogenic differentiation. For example, lncRNA MALA1 promoted the osteogenic differentiation of aortic valve interstitial cells by sponging miR‐204, and miR‐204's target gene, *Smad4*, was up‐regulated.^31^ LncRNA H19 promoted BMSC osteoblast differentiation by targeting miR‐675, and the down‐regulation of miR‐675 subsequently elevated TGF‐β1 mRNA and protein expressions.^32^ LncRNA H19 also functioned as ceRNA of miR‐141 and miR‐22, both of which were negative regulators of osteogenesis.^33^ Through bioinformatics analysis, Gu et al identified 147 lncRNAs totally that were predicted to interact with miRNAs and compete for miRNA binding sites with mRNAs in osteogenic differentiation of periodontal ligament stem cells, revealing the potential lncRNA ceRNA networks.^34^ In hADSCs osteogenic differentiation, the differentially expressed lncRNAs were also identified, and lncRNA H19 was found to significantly influence the expression of osteogenesis‐related genes *ALPL* and *Runx2*.^4^ Our results further confirmed that lncRNAs have a great impact on osteogenic differentiation as their sponging effects on miRNAs could mediate the expressions of osteogenesis‐related mRNAs.

Although the functions of lncRNA PCAT1 in ASC osteogenic differentiation have been clearly investigated in this study, GSEA also identified other differentially expressed lncRNAs. The effects of these lncRNAs on osteogenic differentiation and their interactions with miRNAs and mRNAs remained unclear and require further studies in the future. Additionally, this study lacks of in vivo experiments, which ought to be supplemented to confirm the conclusions.

In summary, lncRNA PCAT1 and *TLR4* were up‐regulated while miR‐145‐5p was down‐regulated in ASC osteogenic differentiation. Additionally, the TLR signalling pathway was activated. PCAT1 and *TLR4* simultaneously targeted miR‐145‐5p targets. PCAT1 promoted the osteogenic differentiation of hADSCs by sponging miR‐145‐5p and up‐regulating *TLR4*.

## CONFLICT OF INTEREST

The authors confirm that there are no conflicts of interest.

## AUTHOR CONTRIBUTION

Lingjia Yu and Hao Qu contributed to research conception and design. Yifeng Yu, Wenjing Li, and Guixing Qiu analysed and interpreted data. Lingjia Yu and drafted the manuscript. Yu Zhao revised the manuscript critically. All authors approved the final manuscript.
