# Supplementary material for: LncRNA‐PCAT1 targeting miR‐145‐5p promotes TLR4‐associated osteogenic differentiation of adipose‐derived stem cells
Source: J Cell Mol Med. 2018 Oct 19;22(12):6134–47. doi: 10.1111/jcmm.13892 (PMC6237555; doi:10.1111/jcmm.13892)
Supplement: Supplementary file 1 [file JCMM-22-6134-s001.docx]

| Genes | Forward (5’-3’) | Reverse (5’-3’) |
| --- | --- | --- |
| PCAT1 | GAGAAGAGAAATCTATTGGAACC | GGTTTGTCTCCGCTGCTTTA |
| miR-145-5p | GTCCAGTTTTCCCAGGAATCCCT | GCTGTCAACATACGCTACGTAACG |
| TLR4 | TCCATTTCAGCTCTGCCTTC | TGGGACACCACAACAATCAC |

**Table S1 Primer Sequences for qRT-PCR**
